# The State of Evidence in Digital Hate Research: An Umbrella Review

**DOI:** 10.1177/00936502251365724

**Published:** 2025-08-28

**Authors:** Jörg Matthes, Kevin Koban, Stephanie Bührer, Thomas Kirchmair, Phelia Weiss, Maryam Khaleghipour, Melanie Saumer, Rinat Meerson

**Affiliations:** 1 University of Vienna, Austria

**Keywords:** digital hate, hate speech, cyberbullying, online harassment, incivility

## Abstract

Digital hate poses a threat to citizens, communities, and societies. Despite numerous studies and reviews on the concept of digital hate, we lack a systematic view of the entire body of scholarship. The aim of this umbrella review is therefore to evaluate the scope, definitions, main findings, and identified gaps of digital hate research. An umbrella review allows one to examine, compare, and evaluate the state of the research across all available reviews in order to point to larger, overarching patterns, shortcomings, and contradictions. We analyzed *N* = 206 narrative, systematic, and meta-analytical reviews. Findings suggest a lack of conceptual clarity, a need to study platform and age differences as well as a need to study digital hate across actors. Also, the analyzed reviews consistently call for experimental, longitudinal, and non-Western, cross-country research. We call for a coordinated cross-disciplinary effort to restructure and harmonize digital hate research.

## Introduction

Social media has become an integral part of people’s daily interactions and activities. Current social media landscapes involve a multiplicity of affordances (Manata & Spottswood, 2022), out of which some enable (and, at times, facilitate) users to live out their most destructive social impulses without much restraint (Brown, 2018). In practice, social media platforms’ unique socio-technical conditions have led to what we intend to establish under the integrative meta-concept *digital hate*. Digital hate is defined intentionally broad as any kind of digitally transmitted malicious expression performed by and directed against an individual or a collective. Accordingly, digital hate involves concepts such as, for instance, cyberbullying (Tokunaga, 2010), online hate speech (Brown, 2017a), or various other forms of harmful speech online (e.g., offensive speech, dehumanizing speech; Paasch-Colberg et al., 2021), on which there is a steady stream of scholarship with hundreds of studies being published in the past years.

Building reliable knowledge around every facet of digital hate to inform public discourse and guide policymakers in a societally advantageous manner can be considered scholarships’ core mission. To be effective, conceptual noise should be avoided as much as possible; however, digital hate research, with its various sub-areas, has largely failed in this regard (e.g., Tokunaga, 2010). Despite large amounts of available evidence across partially overlapping concepts, we lack a systematic view of the entire body of scholarship that accounts for existing noise by taking a meta-level perspective to all available phenomena in order to overcome the current conceptual deadlock. Such a perspective is a necessary first step toward achieving conceptual clarity. Toward that end, an *umbrella review* (i.e., a review of reviews) constitutes a suitable means to compare and contrast meta-insights from a broad research field to “provide an overall examination of the body of information that is available for a given topic, [. . .] highlight whether the evidence base around a topic is consistent or contradictory, and to explore the reasons for the findings” (Aromataris et al., 2015, p. 133). As such, umbrella reviews do not aim at repeating insights of single reviews (Aromataris et al., 2015; Papatheodorou, 2019). Single reviews may be limited because they typically evaluate one specific area of scholarship. Also, sampling criteria can vary from review to review, such that findings may overlap or contradict each other, which makes it difficult to arrive at definitive conclusions (Papatheodorou, 2019). The key advantage of an umbrella review, as the most comprehensive meta-analytical approach available, is that it allows to examine, compare, and evaluate the state of research across *all* available reviews to point to overarching patterns, shortcomings, and contradictions.

This paper engages in such an umbrella review concerning digital hate. We do this by taking an actor-centered approach, meaning we review papers from the perspective of the roles individuals can play in the dissemination of digital hate (i.e., perpetrators, audiences, and targets). This allows us to determine whether designs, conceptualizations, and findings converge or diverge across these actor roles. In addition, we investigate reviews on the entangled detection and contents of digital hate. Overall, this umbrella review seeks to examine and discuss available reviews with respect to (1) definitions and conceptualizations of focal constructs, (2) general characteristics including analyzed platforms, sampling, and author affiliations, (3) main findings, and (4) main research gaps.

## Conceptual Clarity as a Challenge in Digital Hate Research

Social science research has always been troubled by so-called jingle-jangle fallacies, and does so increasingly in recent decades due to, among other reasons like publication incentives, continuing differentiation across academic (sub-)disciplines without superordinate systematic organization being present (see Hanfstingl et al., 2024). While the jingle fallacy refers to distinct constructs or measures that are named identically, the jangle fallacy describes identical constructs or measures established with different terms—with so-called clatters (i.e., similar constructs or measures from distinct paradigms) and clamors (i.e., analogous constructs or measures separately investigated in different fields; Bayer & LaRose, 2018) worsening issues across disciplinary lines. Specifically, Lawson and Robins (2021) detailed 10 “sibling relationships between two (or more) constructs” (p. 346) ranging from conceptual and empirical overlaps to shared trajectories and underlying processes.

In research on digital hate, it is evident that the field is driven by a few established and elaborated constructs. Most notably, lots of research engages online hate speech as its central concept without agreeing on a univocal definition or even definitional approach (Sellars, 2016), with scholars like Brown (2017b) even arguing against compositional definitions and for seeing it merely as “a heterogeneous collection of phenomena held together by family resemblances” (p. 610). Another key concept that has been differentiated from online hate speech primarily via conduct repetitiveness and (sometimes) a more defined perpetrator-victim relationship is cyberbullying (Rudnicki et al., 2023). For cyberbullying, most studies follow Olweus (1993) well-established core criteria of traditional bullying (i.e., aggression, harm, intentionality, etc.) and adapt it toward digital contexts by further specifying the means or medium via which cyberbullying is conducted and affordances that may facilitate it (see Olweus & Limber, 2018). However, similar to online hate speech, cyberbullying has been considered challenging to define univocally, resulting in an absence of an agreed-upon definition (e.g., Vismara et al., 2022).

Besides online hate speech and cyberbullying, online incivility has been another variably conceptualized focal point in the literature, particularly in work on malicious political communication (Chen et al., 2019). Conceptual work involves discussions where incivility is distinguished from impoliteness and intolerance (Rossini, 2022) and further differentiated, for instance, into personal-level and public-level incivility or utterance, discursive, and deceptive incivility (Stryker et al., 2016). Although all these different terms are conceptually grounded in a variably set distinction between violations of conversational, social, democratic, and safety norms, advanced conceptual clarity has again been called for on regular basis (see Chen et al., 2019; Rossini, 2022).

In addition to these fairly established key constructs, digital hate literature also comprises several other concepts (e.g., cyber-/online aggression, harassment, hostility, violence) and almost countless more or less subtly specified construct extensions and hybrids, including several kinds of action (e.g., cyber-/online [dating or partner] abuse/victimization, stalking, toxicity, trolling, or different group-directed phobias and isms) and ways of speech (e.g., offensive, dangerous, harmful, violent, abusive, or aggressive online speech). Importantly, most of these concepts can come with marginally different foci and slightly dissimilar (and often variable) definitions.

Altogether, this brief overview of the conceptual state of digital hate research points toward a significant issue: That is, this field is populated by numerous variably defined and conceptualized constructs of interest of presumably substantial (based on family resemblances) yet somewhat arbitrarily determined (given said definitional and conceptual ambiguities) overlap. In other words, we posit that digital hate research is just another field with notable jingle-jangle issues. Because of this, it appears questionable whether current digital hate scholarship can sufficiently fulfill its societal function to gain reliable and valid knowledge that can be used to improve public education (e.g., by informing debates) and guide policy decisions (e.g., by advising policymakers). A systematic “diagnosis” of digital hate research as a whole is necessary to form the basis for a more unitary approach. Although we acknowledge that such *lumping* may come with the risk of ignoring relevant conceptual nuances (Lawson & Robins, 2021), we consider it a necessary step for addressing conceptual clarity problems before meaningful *splitting* can be undertaken to advance the current conceptual state.

## Objectives of the Present Study

As detailed above, digital hate is defined broadly by design to allow for incorporating many discrete concepts. While study-level reviews are inherently limited to single or a few constructs and may involve lots of conceptual idiosyncrasies originating from ad-hoc definitions specifically adapted to a particular study at hand, an umbrella review might not only enable a cross-conceptual overview of definitional criteria but also benefit from reviews’ need for at least somewhat standardized conceptualizations. Accordingly, we ask:

**RQ1:** How are different facets of digital hate defined and conceptualized across digital hate reviews?

Digital hate does occur to varying extents across digital information and communication platforms (Van Duyn & Muddiman, 2022). Nevertheless, the terminology used within the digital hate literature (e.g., cyber, online, internet) implicitly suggests that the internet and social media platforms are monoliths, despite the numerous contextual factors that should be considered (e.g., Pelletier et al., 2020). These platforms are instead situated within a competitive market in which any niche is fought over, which leads to constant alternations of innovation, fragmentation, and hybridization. Accordingly, platforms differ with respect to primary features and affordances, dominant user groups, practices, shared meanings, as well as platform-determined and socially negotiated rules and policies, out of which each may be subject to dynamic transformation and may individually or in interaction favor the occurrence of digital hate in general or particular manifestations of it (Cinelli et al., 2021). For example, the discourses within the incel community permit and facilitate such expression (Tranchese & Sugiura, 2021). Anecdotally, some platforms are notorious for digital hate (e.g., Telegram, Gab, or 8Chan). Although cross-platform research, which may provide systematic insights into patterns, is challenging (i.e., questions of equivalence, Schemer & Reiners, 2023), studies have shown differences in both hateful language (e.g., Phadke & Mitra, 2020) as well as its perception (Sude & Dvir-Gvirsman, 2023). Cross-platform research on digital hate is crucial for understanding its spread, evolution, and impact across various digital environments, assessing the efficacy of platform-specific moderation policies, identifying interconnected hate networks, and informing comprehensive counter-strategies and policy measures. Reviews may be particularly useful to shed light on cross-platform similarities and divergences in a more comprehensive manner given their aggregating nature. Umbrella reviews may allow for additional meta-level conclusions on platform idiosyncrasies and could serve to identify (in-)consistencies across constructs. Thus, we ask:

**RQ2a:** (How) Is the role of platforms accounted for across digital hate reviews?

Concerning (online) media, prominent theoretical approaches (e.g., the differential susceptibility to media effects model; Valkenburg & Peter, 2013) have proposed that developmental levels may not only act as predictors of media-related preferences but also as moderators for how individuals respond to content exposure. Traditionally, developmental approaches have placed a strong emphasis on younger age groups, ranging from toddlers to young adults, which is reflected by popular literature’s buzz on colloquialisms like Gen Z(oom) or Gen Alpha. Recent large-scale studies substantiate this emphasis, documenting the strongest negative relationships between social media use and psychological well-being during early and late adolescence for women and mid- and late adolescence for men (Orben et al., 2022). However, digital hate affects people of all ages (Barlett & Chamberlin, 2017), but the expressions, mechanisms, and challenges can vary across age groups. For example, adults may be more involved in workplace cyberbullying (Kowalski et al., 2018) or spreading politically motivated hostility (Bor & Petersen, 2022) compared to younger people. Examining digital hate across the lifespan is important for understanding how age influences experiences and outcomes, offering insights into protective factors and interventions across different life stages. How age groups vary across concepts can be best answered through an umbrella review. Thus, we ask:

**RQ2b:** What age groups are considered across digital hate reviews?

A sweeping dominance of White scholars from the Global North has long been evidenced in academia, including in communication science (e.g., Freelon et al., 2023). As such work reveals, the hegemonic standing of White American and White Western European scholarship within the Anglophone communication science community can be documented reliably across various criteria, such as high-impact publication rates, overall citation counts, or editorial leadership positions, and closely reflects a stable overreliance on empirical evidence from so-called WEIRD (i.e., Western, Educated, Industrialized, Rich, Democratic) samples (Henrich et al., 2010). Concerning these biases, umbrella reviews may provide a novel viewpoint due to reviews being their unit of analysis. In other words, a meta-perspective, for instance, on authors’ current affiliations, could be particularly insightful because it may be another neglected indicator of academic centrality (see Demeter, 2017). Apart from that, it might also be plausible that peripheral scholarship is more strongly represented among reviews, given that systematic aggregations of non-English samples may overwrite disadvantageous publishing biases. Thus, we ask:

**RQ2c:** (How) Do WEIRD biases exist in terms of authorship of digital hate reviews?

As outlined above, digital hate research covers an immense heterogeneity of concepts. This heterogeneity makes it challenging to synthesize findings. As a solution for this umbrella review, we suggest formally separating the triad of actors that are primarily involved in digital hate: perpetrators, audiences, and targets. Scholars have analyzed interactions between motives of *perpetrators* and situational circumstances, pointing to aspects like perceived outgroup threat or anonymity of digital environments (e.g., Suler, 2004). For the second question, research has primarily explored interventions to prevent or change perpetrators’ hateful conduct (e.g., Barlett et al., 2018). The *audience*, defined as “witnesses” who are neither directly associated with the perpetrator(s) nor with the target(s) of an act of digital hate, plays a crucial role in this context. Audience members may act along an action continuum ranging from apathetic bystanders to highly engaged defenders. Research has examined factors explaining, for example, why some audience members intervene and counteract while others do not (e.g., Kalch & Naab, 2017). In addition to perpetrators and bystanders, there are several studies investigating how *targets* of digital hate acts are affected by it with respect to psychological, social, and behavioral consequences (e.g., Zempi, 2014).

We understand digital hate content and detection as intersecting categories. The intersection of digital hate content and its detection arises from the growing need for advanced algorithms to effectively identify and mitigate harmful online material (i.e., by incorporating specific linguistic features; see Wiegand et al., 2022). By doing so, we may be able to illuminate the state of research about who is involved in what way when digital hate occurs, including cross-connections between actors (e.g., whether research on perpetrators is tied to audience research or whether audience research is connected to target research). Lacking cross-connections between actors may jeopardize conceptual harmonization and clarity. If, for instance, research is separately reviewed for targets, audiences, and perpetrators, overarching patterns involving similarities and contradictions cannot be determined. Similarly, comparing meta-analytical evidence regarding perpetrators, audiences, and targets may enable us to identify literature gaps. We ask:

**RQ3a:** What are the main findings (with respect to perpetrators, audiences, targets, and contents) across digital hate reviews?
**RQ3b:** What is the state of meta-analytic evidence (with respect to perpetrators, audiences, targets, and contents) in the field of digital hate?

Finally, it is one of the most pivotal tasks of reviews to identify research gaps and outline future research avenues. While it may be considered obvious that many notable gaps differ across actors, an isolated perspective on perpetrators, audiences, targets, and contents may still miss out on potential synergies (or lack thereof). For example, a particular lack of evidence identified for one of them may have remained unnoticed so far for the others. Providing a comprehensive overview of research gaps can inform us about the state of the entire field and may speak to the scope, validity, and generalizability of available findings. It is, therefore, essential to systematically assess them together for actors and contents. Thus:

**RQ3c:** What are the main research gaps (with respect to perpetrators, audiences, targets, and contents) identified across digital hate reviews?

## Method

### Sampling

Following the Preferred Reporting Items for Systematic Reviews and Meta-Analyses (PRISMA) statement (Page et al., 2021), we first conducted a literature search in Google Scholar to gain an initial overview that resulted in 51 relevant records. We then conducted a systematic search on Web of Science (WoS) to identify all available reviews, meta-analyses, or other types of syntheses of empirical studies. This WoS search was later complemented by a Scopus search, as a combination of both databases is said to provide the best results (Bramer et al., 2017). Our search strategy aimed to combine variants of these three components: (1) digital, (2) hate, and (3) review. For each component, as many keywords as possible were identified and linked using the logical operator “OR” (see Supplemental Table A1 in the Online Appendix for a detailed list: https://osf.io/ya456/.

Asterisks were applied to cover different types of spelling. These three blocks of keywords were then concatenated with “AND” in the first search string. A second search string was necessary because some “hate” terms imply an online setting (e.g., deepfake or trolling), such that it is unlikely that authors would use them in combination with synonyms for “digital.” The following additional constraints were set: (1) Given the extensive list of terms, only titles were screened for keywords. (2) Only reviews, meta-analyses, and other types of syntheses published in English were considered (i.e., other languages were excluded from the search). (3) WoS categories were limited to research areas relevant to this umbrella review’s scope, such as social sciences, communication, psychology, and computer sciences. Irrelevant categories such as chemistry were excluded (see Supplemental Table A2, Online Appendix).

**Figure 1.**
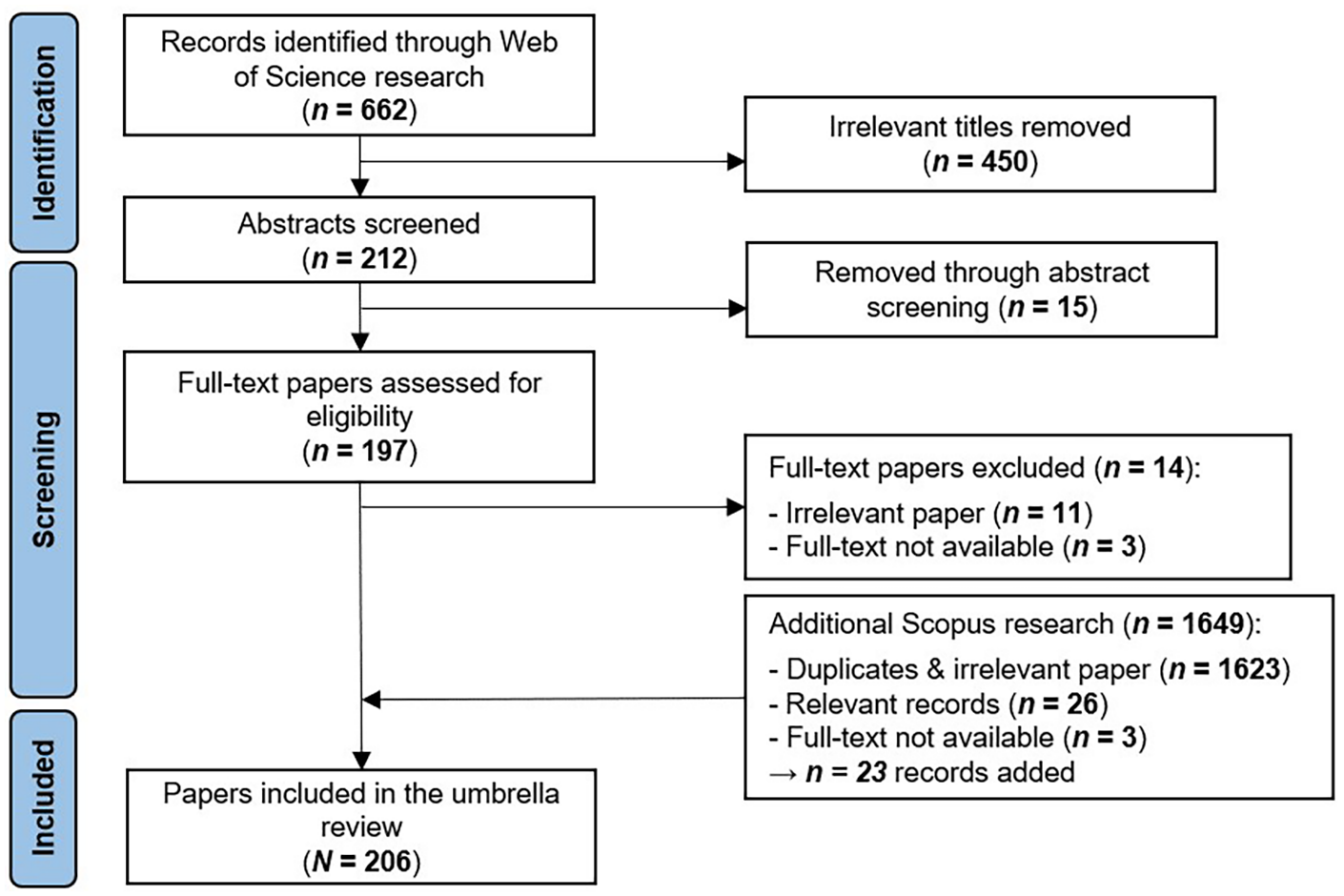
PRISMA flow diagram for all stages of the literature search.

The WoS search string (which can be retrieved from Supplemental Table A3 on OSF) was executed on March 8, 2023 and returned 662 records that needed to be screened. No additional restrictions were set on the publication date. Furthermore, all types of contributions (i.e., articles, book chapters, early access, proceedings) and all age groups were considered. For umbrella reviews focusing on narrow constructs and effects (e.g., randomized control trials), it is common to include only systematic reviews and meta-analyses. Given the broad scope of digital hate and the aim of providing a comprehensive overview, this paper also includes narrative reviews, similar to the approach by Valkenburg et al. (2022). However, we document all findings for each paper type on OSF. Records were excluded if they were not identified as some kind of review, meta-analysis, or another type of study synthesis or if they did not address digital hate. The titles of 662 extracted documents were screened by two trained coders (see Figure 1). The most frequent excluded topics were (1) digital interventions for offline issues, (2) education content unrelated to digital hate, (3) child maltreatment or child abuse, (4) fraud victimization, (5) (negative) online reviews of products, hotels, etc., (6) study protocols, (7) cybersecurity threats, or (8) not hateful online behaviors. Overall, 450 records were dropped via title screening, leaving 212 documents for abstract screening. After two trained coders screened the remaining abstracts, 15 additional documents were excluded based on topical irrelevance. The subsequent full-text reading revealed 11 more irrelevant papers. For three documents, full-text was not available, resulting in a WoS sample of 183 records. All records from the preliminary Google Scholar literature search were identified through the WoS search. The complementary Scopus search (see Supplemental Table A4 on OSF to retrieve the search string) was performed on August 1, 2023. WoS keywords and criteria were used and only reviews published until March 8, 2023 were considered. Of the 1,649 records found, 1,623 were either duplicates or thematically irrelevant. For three papers, full text could not be obtained. After including the remaining 23 records from the Scopus research, the final sample of documents to be coded was *N* = 206.

### Quantitative and Qualitative Coding

All 206 identified records were examined quantitatively and qualitatively. A comprehensive overview of category operationalizations can be found in the codebook on OSF. The codebook is divided into two parts. In the first part, 14 categories were analyzed quantitatively: year, method, main concept(s), language scope, number of included papers, age (divided into children, adolescents, young adults, and adults), actors (divided into perpetrators, audiences, and targets), content, and platform. Four categories were examined qualitatively: geographical allocation of the authors’ affiliated institution, definition of the main construct(s), main result(s), and main research gap(s). Table 1 provides a brief description of these quantitative and qualitative categories. The second part addresses which categories of variables have been examined. Thirteen categories were established: (1) conceptualization and measurement, (2) detection, (3) prevalence, (4) offline hate, (5) sociodemographics, (6) attitudes and beliefs, (7) media use and virtual environment, (8) mental and physical health, (9) prevention and intervention, (10) school and work, (11) skills and competences, (12) social factors, and (13) traits, cognition, and emotion. Additionally, effect sizes for main effects were extracted from meta-analyses to provide deeper insights into meta-analytical evidence.

**Table 1.** Description of the Analyzed Quantitative and Qualitative Categories.

Category	Description
Year (QT)	Year of publication was coded.
Method (QT)	Records were assigned to one of four categories: (1) bibliometric/ scientometric analysis, (2) narrative review/ synthesis, (3) systematic/ scoping review, or (4) meta-analysis.
Main concept(s) (QT)	Main concept(s) were inductively coded using paper titles, resulting in 18 categories: (1) cyber/digital/online dating/intimate partner abuse/violence/victimization, (2) cyber/online abuse, (3) cyber/ online hate, (4) cyberaggression, (5) cyberbullying, (6) cyberbystander, (7) cyberracism, (8) cyberstalking, (9) cyberharassment, (10) cybervictimization, (11) cyberviolence, (12) deepfake, (13) digital threat, (14) extremism/ terrorism/ radicalization, (15) hate speech, (16) incivility, (17) social media based violence, (18) technology-facilitated domestic/dating/ sexual/intimate partner abuse/violence.
Language scope (QT)	Records were coded on whether authors included only English studies, allowed other languages (coded “English+”), or provided no information.
Included papers (QT)	Number of papers included in bibliometric or scientometric analyses, systematic or scoping reviews as well as meta-analyses was coded.
Age (QT)	Multiple dichotomous yes/no responses were used to code whether children, adolescents, young adults and/or adults were sampled.
Actors (QT)	Dichotomous yes/no responses were used to code whether reviews focused on perpetrators, audiences and/or targets.
Content (QT)	Dichotomous yes/no responses were used to code whether reviews focused on the content of digital hate.
Platforms (QT)	Dichotomous yes/no responses were used to code whether different/ specific social media platforms/applications were analyzed.
Geographical allocation (QL)	Countries in which author institutions are located were first coded qualitatively and then summarized for quantitative analyses.
Definition(s) (QL)	Definition(s) of main concept(s) was/were extracted in full sentences.
Main result(s) (QL)	Main result(s) was/were extracted in full sentences.
Main gap(s) (QL)	Main research gap(s) was/were extracted in full sentences.

*Note*. QT = quantitatively coded; QL = qualitatively coded.

Two trained coders evaluated 25 randomly selected documents (12.1% of the sample). This first coding resulted in sufficient agreement (Krippendorf’s α ≥ .8) in 21 of the 27 categories (see OSF Supplemental Table A5). Concerning the unreliable categories, both coders resolved ambiguities through discussion and adjusted the codebook accordingly. Subsequently, both authors again coded 25 randomly selected documents with respect to the previously unreliable categories. Krippendorf’s α was calculated again, and all categories scored above .8. Finally, one author coded the entire sample (see codebook and Supplemental Tables A6–A9 on OSF).

## Results

An overview of all quantitative results can be found in Table 2 (additionally, Supplemental Tables A10–A13 on OSF show these results categorized by paper type). The oldest review dates from 2009, and until 2013, a maximum of three reviews were published per year. From 2014 (*n* = 10) on, the number of reviews slowly increased, with a peak in 2022 (*n* = 50). About half of the included reviews were systematic or scoping reviews (*n* = 106; 51.5%), followed by narrative reviews or syntheses (*n* = 52; 25.2%), meta-analyses (*n* = 39; 18.9%), and bibliometric and scientometric analyses (*n* = 9; 4.4%). The latter included the largest number of papers (*M* = 1,487, *SD* = 1,076, range: 321–3,371), while systematic or scoping reviews comprised slightly more papers (*M* = 45, *SD* = 45, range: 4–266) than meta-analyses (*M* = 36, *SD* = 29, range: 2–131).

**Table 2.** Quantitative Codings with Multiple Categories.

Category	Occurrence (*N* = 206)
Year	2009 (*n* = 1; <1%); 2010 (*n* = 2; 1%); 2,012 (*n* = 3; 1%); 2013 (*n* = 3; 1%); 2014 (*n* = 10; 5%); 2015 (*n* = 16; 8%); 2016 (*n* = 10; 5%); 2017 (*n* = 8; 4%); 2018 (*n* = 21; 10%); 2019 (*n* = 18; 9%); 2020 (*n* = 23; 11%); 2021 (*n* = 33; 16%); 2022 (*n* = 50; 24%); 2023 (*n* = 8; 4%)
Method	Bibliometric/scientometric analysis (*n* = 9; 4%); narrative review/synthesis (*n* = 52; 25%); systematic/scoping review (*n* = 106; 51%); meta-analysis (*n* = 39; 19%)
Main concept	Cyber/digital/online dating/intimate partner abuse/violence/victimization (*n* = 9; 4%); cyber/online abuse (*n* = 2; 1%); cyber/online hate (*n* = 6; 3%); cyberaggression (*n* = 3; 1%); cyberbullying (*n* = 127; 62%); cyberbystander (*n* = 5; 2%); cyberracism (*n* = 1; <1%); cyberstalking (*n* = 4; 2%); cyberharassment (*n* = 1; <1%); cybervictimization (*n* = 11; 5%); cyberviolence (*n* = 3; 1%); deepfake (*n* = 4; 2%); digital threat (*n* = 1; <1%); extremism/ terrorism/ radicalization (*n* = 8; 4%); hate speech (*n* = 19; 9%); incivility (*n* = 1; <1%); social media based violence (*n* = 2; 1%); technology-facilitated domestic/ dating/ sexual/ intimate partner abuse/ violence (*n* = 10; 5%); trolling (*n* = 1; <1%)
Language scope	English papers only (*n* = 75; 36%); English and other languages (*n* = 49; 24%); no information available (*n* = 82; 40%)
Included papers	Bibliometric or scientometric analysis (*M* = 1,487, *SD* = 1,076); systematic or scoping review (*M* = 45, *SD* = 45); meta-analysis (*M* = 36, *SD* = 29)
Age	Children (*n* = 66; 32%); adolescents (n = 129; 63%); young adults (*n* = 62; 30%); adults (*n* = 31; 15%)
Actors	Perpetrators (*n* = 127; 62%); audiences (*n* = 32; 16%); targets (*n* = 133; 65%)/perpetrators & targets (*n* = 108; 52%); perpetrators & audiences (*n* = 23; 11%); targets & audiences (*n* = 23; 11%); ≥ two actors (*n* = 112; 54%); three actors (*n* = 21; 10%)
Geographical allocation	Australia (*n* = 9; 4%); Austria (*n* = 1; <1%); Belgium (*n* = 2; 1%); Brazil (*n* = 2; 1%); Canada (n = 8; 4%); Chile (*n* = 1; <1%); China (*n* = 5; 2%); Colombia (*n* = 3; 1%); Cyprus (*n* = 1; <1%); Ecuador (*n* = 1; <1%); Egypt (*n* = 1; <1%); Greece (*n* = 2; 1%); Hong Kong (*n* = 2; 1%); India (*n* = 11; 5%); Indonesia (*n* = 3; 1%); Ireland (*n* = 1; <1%); Italy (*n* = 5; 2%); Jordan (*n* = 1; <1%); Kazakhstan (*n* = 1; <1%); Kenya (*n* = 1; <1%); Malaysia (*n* = 7; 3%); Mexico (*n* = 1; <1%); Netherlands (*n* = 1; <1%); New Zealand (*n* = 1; <1%); Pakistan (*n* = 1; <1%); Portugal (*n* = 4; 2%); Romania (*n* = 3; 1%); Saudi Arabia (*n* = 3; 1%); Singapore (*n* = 3; 1%); South Africa (*n* = 2; 1%); Spain (*n* = 22; 11%); Sweden (*n* = 1; <1%); Switzerland (*n* = 3; 1%); Turkey (*n* = 2; 1%); UK (*n* = 9; 4%); USA (*n* = 33; 16%); Vietnam (*n* = 1; <1%); multinational (*n* = 48; 23%)

RQ1 aimed for an overview of definitions and conceptualizations of digital hate. Due to the lack of a universally accepted typology, we approached this in a bottom-up manner. In the first step, we quantitatively summarized review prevalence across different concepts, followed by a second step, where we qualitatively analyzed definitional practices and criteria. When merely looking at quantities, cyberbullying accounted for the (relative) majority of reviews (*n* = 127; 61.7%), followed by online hate speech (*n* = 19; 9.2%), cybervictimization (*n* = 11, 5.3%), and cyber-/online hate (*n* = 6, 2.9%). Under slightly varying wordings, digital hate against (potential) romantic partners (*n* = 19, 9.2%) and online extremism (*n* = 8, 3.9%) also emerge as focal points. In addition to these frequently reviewed concepts, our sample comprised several distinctly worded topical foci that have been reviewed only a few times (see Table 2; a word cloud visualizing this distribution is available in Supplemental Figure A3 on OSF).

Focusing on how our reviews conceptualize those different digital hate facets, cyberbullying occupies a somewhat exceptional status. That is, many (albeit not all) reviews concur in several defining features (i.e., intention, repetitiveness, power imbalance between perpetrators and targets, aggressiveness, generally harmful nature, and, to some extent, target defenselessness), variabilities (i.e., directness/overtness, medium/platform, perpetrator and target characteristics, modality), and facilitating technology affordances (i.e., anonymity, pervasiveness, ubiquity, audience reachability, convenience), which can likely be explained (at least partially) by some work on (cyber-)bullying being considered canonical (most notably, Olweus, 1993; Smith et al., 2008). Apart from these broad agreements, review conceptualizations nevertheless exhibited a long list of vaguely defined phenomenological specifications (e.g., hostility, threats, abusiveness, harassment, impersonation, imitation, manipulation, denigration, embarrassment, intimidation, etc.) that may “creep” into otherwise straightforward definitions and create substantial overlap with other digital hate concepts, as well as other conceptual extensions (e.g., differentiations between various subtypes of harm) that may produce semantic dilution (see Haslam, 2016). A similarly broad yet fairly one-dimensional agreement can be identified among reviews about online hate speech, cyber-/online hate, and cyberracism where a considerable number of (but not nearly all) conceptualizations involve some notion of systematically disadvantaged or societally vulnerable targets (due to gender, gender identity, sexual orientation, race, origin, descent, color, nationality, ethnicity, caste, religion, socioeconomic status, marital status, political affiliation, marital status, health status, disability, physical appearance) as a defining feature (see Gelber, 2021). However, beyond this agreed-upon conceptual core, definitions vary substantially in what they consider an expression of hate. These expressions range from mild forms like microaggressions, offensiveness, and disrespect to more severe manifestations such as threatening language and mass instigation, which themselves can be ambiguously defined (Windisch et al., 2022).

Except for these agreements within reviews on both cyberbullying and online hate (speech), reviews across digital hate concepts primarily offer what Brown (2017b) characterized as complex disjunctive definitions: Presumably exhaustive lists of phenomena with an unspecified shared essence that may never be truly definitive so much so that the “most ordinary language users would instinctively” (p. 565) find yet another, still missing phenomenon. Save clustered emphases on features closely related to romantic or sexual relationships, audiences (evident mainly in cyber-bystander reviews), or advanced technologies (e.g., deepfakes), definitions across supposedly discrete concepts hence not only overlap significantly with each other (typically even more than within concepts) but also appear, at least to some extent, arbitrarily worded and, thus, somewhat interchangeable.

To gain quantifiable insights into these conceptual issues, we examined the degree of overlap in references across our meta-analyses subsample (excluding Gilbar et al., 2023 where references could not be identified with necessary certainty) as the most systematically conducted and transparently documented review type following the corrected covered area (CCA) method by Hennessy and Johnson (2020). As expected, the full reference matrix (*N* = 945 unique references across *N* = 38 meta-analyses; Excel spreadsheet and R-code are available on OSF) resulted in a very low CCA of .016, indicating very heterogeneous primary studies. Similar results were found when only meta-analyses about (cyber)bullying were considered (*n* = 26; CCA = 0.023). Pairwise cluster identification allows for a more meaningful evaluation. Here, we identified the greatest overlap between Sun et al. (2016), who reviewed gender differences in cyberbullying perpetration, and Sun and Fan (2018), where gender differences in cyber-victimization are addressed (CCA = 0.549). Although still with an at best small-to-moderate overlap, noteworthy CCAs could be found for meta-analyses on prevention and intervention of cyberbullying (Gaffney et al., 2019; Lan et al., 2022; E. D. Ng et al., 2022; Wang & Jiang, 2023), which each referred to a relatively small pool of primary studies (*n*s = 9–48; CCAs = 0.102–0.350), on mental health issues related to cyberbullying and cybervictimization (Fisher et al., 2016; Gini et al., 2018; John et al., 2018; Li et al., 2024; Tran et al., 2023), where each used a slightly greater pool of primary studies (*n*s = 17–55; CCAs = 0.029–0.135), and on general predictors, again, of cyberbullying and cyber-victimization (Barlett & Coyne, 2014; Chen et al., 2017; Marciano et al., 2020; Sun et al., 2016; Sun & Fan, 2018), in which larger pools of primary studies were covered each (*n*s = 56–131; CCAs = 0.038–0.549). In sum, these small-to-moderate overlaps indicate a few somewhat robust sub-topics, especially in the cyberbullying literature within an otherwise highly diversified and conceptually discordant broader field.

RQ2a asked how different platforms are considered across reviews. Less than a fifth (21.8%) of all sampled reviews provided information on platform differences, and only a single review addressed platform comparisons as a major topic. Here, Matamoros-Fernández and Farkas (2021) concluded that Twitter (54.8%) was the most studied platform in their sample, followed by Facebook (34.6%). Within incivility research, Twitter and Facebook also play a more important role than other platforms (Ng et al., 2020). Moreover, especially when it comes to hate speech detection, Twitter has frequently been used as a data source in the past (e.g., Aldera et al., 2021). Apart from these limited insights, notable findings on cross-platform comparisons are absent across reviews, answering RQ2a.

RQ2b asked about age groups. Adolescents were the most frequent group (*n* = 129; 62.6%), followed by children (*n* = 66; 32.0%), young adults (*n* = 62; 30.1%), and adults (*n* = 31; 15.0%) (see Supplemental Figure A1 on OSF for an overview across paper types). Interestingly, all 66 reviews that included children studied them together with adolescents, suggesting that children are less well-represented than it might seem. This would then reflect the fact that several authors pointed out that findings on children are scarce in this field (e.g., Chen et al., 2017; Gini et al., 2018). The comparatively low number of adult samples corresponds with existing calls for studies with older samples (e.g., Kim & Ferraresso, 2023).

RQ2c asked about WEIRD biases in reviews on digital hate. Henrich et al. (2010) stated that they use the term “Western” to refer to Northwestern Europe (e.g., UK, France, Germany), the United States, Canada, New Zealand, and Australia, which we therefore clustered as “Western.” Overall, 38.3% of the reviews were published by authors affiliated only with Western institutions (*n* = 79). Additionally, researchers affiliated with Western institutions were at least co-authors in 26 papers (12.6%), leaving 49.0% exclusively by non-Western affiliated authors (*n* = 101). Authors affiliated with higher education institutions from Southern European countries such as Spain, Greece, and Portugal, which are by definition part of the non-Western cluster, constitute another 14.1% (*n* = 29) of the sample. Especially Spain appears highly represented (*n* = 22; 10.7%). Authors from South and East Asian institutions account for 17.5% (*n* = 36), with Indian authors (*n* = 11; 5.3%) being most prominent in hate speech detection research. The noteworthy overrepresentation of this computer science-oriented subfield in this cluster also means a lack of reviews from other disciplinary perspectives (e.g., social sciences) by researchers affiliated with Asian institutions. Reviews published exclusively by authors affiliated with Latin American (*n* = 8; 3.9%) or African institutions (*n* = 5; 2.4%) were less common (see Saleem et al., 2022). Also, 36.4% of sampled reviews solely included papers written in English (*n* = 75), 39.8% did not report which language they sampled (*n* = 82), and only 23.8% stated that they included non-English papers (*n* = 49). Although an adequate baseline for non-Western authors cannot be defined a priori, the findings suggest a WEIRD bias with respect to authorship. An overview of WEIRD bias across paper types is available in Supplemental Figure A2 on OSF, illustrating that this bias is slightly more pronounced in systematic and scoping reviews, as well as meta-analyses, compared to narrative reviews.

**Figure 2.**
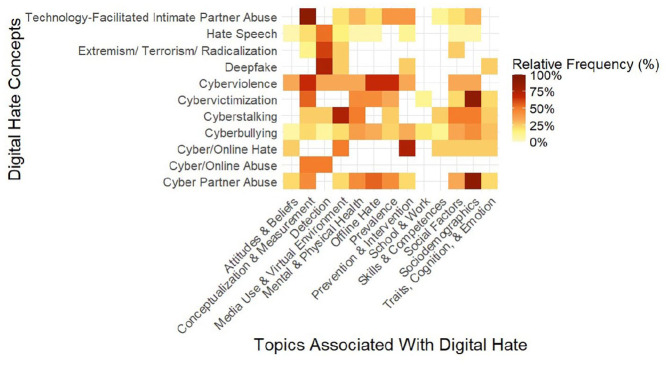
Heatmap of research topics across digital hate concepts. *Note*. Categories represented by fewer than three cases were omitted from this visualization.

In RQ3a and RQ3b, we explored the main findings across digital hate reviews and examined the state of the meta-analytical evidence. Although digital hate is a prevalent phenomenon globally, substantial variation exists across age groups, gender identifications, educational backgrounds, and countries (e.g., Camerini et al., 2020). Victimization and perpetration prevalence rates vary widely across studies, sometimes exceeding 70% (e.g., Wilson et al., 2023), primarily due to differing conceptualizations of digital hate and varying measurements. Reviewing meta-analyses on prevalence indicates that, on average, around 10% of individuals experience perpetration or victimization (see forest plots in Supplemental Figures A4 and A5 on OSF for an overview). However, the heat map in Figure 2 highlights that prevalence data is primarily available for cyberbullying and digital hate related to dating, while information on other concepts, such as hate speech or deepfakes, remains scarce.

Topics and variables associated with digital hate show notable variation across different concepts, as illustrated in Figure 2, with cyberbullying standing out by covering all thematic areas due to the comparatively large number of studies available. The first topic, attitudes and beliefs, is rarely studied across digital hate concepts. In contrast, discussions around conceptualization and measurement methods are frequent, though they are less common for some concepts, such as hate speech. Conversely, many studies on hate speech focus on detection methods, while detection is rarely a focus in dating-related digital hate research. Media use and virtual environment variables appear across most digital hate concepts to some extent, while studies linking digital hate to offline behaviors are largely concentrated in cyberbullying or dating-related research, with minimal exploration in areas like hate speech or online hate. Prevention and intervention methods are commonly addressed in papers on cyberbullying and dating-related digital hate, with some coverage in online hate and hate speech studies, but are notably absent in research on cyberstalking or extremism. As expected, school and work-related variables predominantly appear in studies on cyberbullying and are rarely addressed in other contexts. Additionally, social factors and sociodemographic variables are commonly reviewed in studies on cyberbullying and dating-related digital hate but are rarely explored in research on hate speech or deepfakes. Finally, traits, cognition, and emotions are addressed in some studies on cyberbullying and dating-related digital hate, but not for hate speech or extremism.

Exploring the individual actors of digital hate in more detail, starting with perpetrators, scholars consistently reviewed factors that correlate with the likelihood of engaging in perpetration. Personal characteristics such as male gender, low academic performance, substance use, frequent information technology use, prior experiences of others being bullied, low levels of self-control, tendencies toward moral disengagement, or antisocial personality traits (i.e., low empathy) have consistently been reported as predictors of hateful online behaviors. An overview of all variables associated with perpetrators in meta-analyses, sorted by overarching categories like sociodemographics or social factors, can be found in the forest plots on OSF (Supplemental Figures A6–A13). Given the large number of variables, only selected findings are highlighted here as examples. While (anti-)social traits play significant roles in perpetration research and demonstrate considerable effect sizes (e.g., Lo Cricchio et al., 2021; Supplemental Figure A7), findings on emotional factors are limited. Additionally, mental and physical health are frequently studied as meaningful predictors and outcomes (e.g., Fulantelli et al., 2022; Supplemental Figure A8). Notably, there is evidence of an interrelationship between perpetration and victimization (Lozano-Blasco et al., 2020), suggesting that being victimized by digital hate may predict perpetration (e.g., Estévez et al., 2020). This overlap between roles may be one reason for the associations found between perpetration and health-related variables. Further, although media use appears to play an important role in perpetration (Supplemental Figure A9), impacts of specific platforms and their affordances remain unclear, especially for concepts beyond cyberbullying. Factors related to the school environment, as well as social and family influences, have also been found to play a critical role (e.g., Zych et al., 2019; Supplemental Figures A10–A11). Additionally, meta-analytic evidence shows strong associations between digital and offline hate perpetration (Supplemental Figure A12). Lastly, findings indicate that digital hate can be prevented through methods such as serious video games (e.g., Calvo-Morata et al., 2020) or school-based interventions (i.e., creating school norms; e.g., Davis et al., 2014; Supplemental Figure A13). Notably, most of this work focused on cyberbullying.

As for audiences, reviews addressed factors that encourage desirable bystander activities (e.g., Lambe et al., 2019). Personal characteristics such as gender (i.e., women being more likely to act than men), traits (e.g., empathy, resistance to group pressure), first-hand experiences with digital hate, popularity, close relationships to targets, as well as situational factors such as perceived severity of the hateful act or other bystanders’ visible (or imagined) behavior have been found to help explaining why some individuals counteract digital hate while others do not (e.g., Domínguez-Hernández et al., 2018; Ma et al., 2019). However, the consequences for those who decide on countering digital hate are less well understood. Some reviews have also looked into intervention effects and revealed that they can be effective (e.g., Gaffney et al., 2019; Windisch et al., 2022). From a meta-analytical perspective (see OSF Supplemental Figure A14), research on concepts beyond cyberbullying remains limited, particularly regarding emotions, personality traits, media use, environmental factors, and offline correlates. In addition to moral reasoning, social factors (such as group pressure) appear to play an important role in motivating intervention, as indicated by effect sizes.

**Figure 3.**
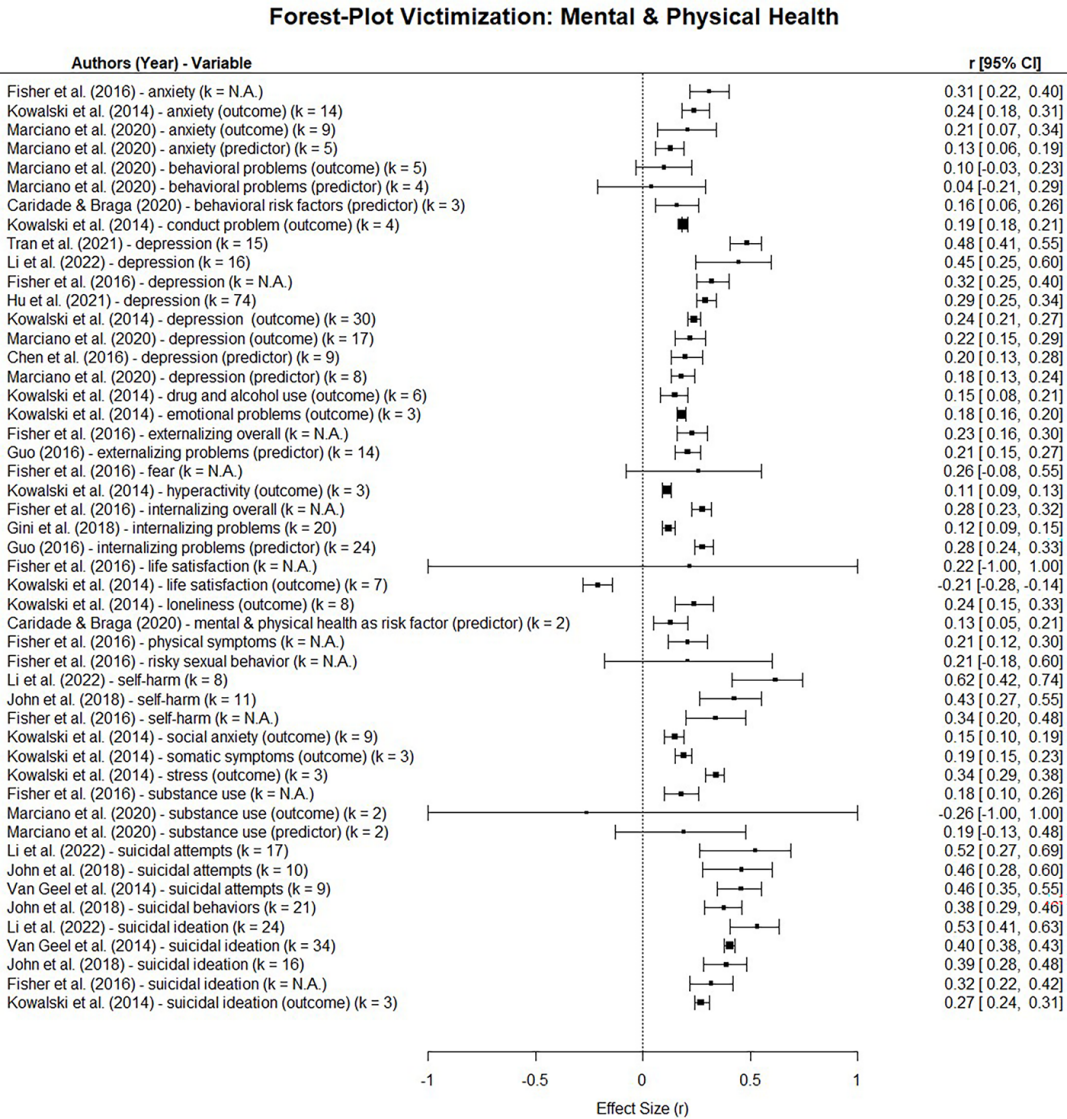
Forest plot of meta-analyses on relations between mental & physical health and digital hate victimization.

When it comes to targets, the serious harms associated with being victimized by digital hate are well documented (e.g., Fernet et al., 2019). These include a wide range of health-related outcomes, which are visually summarized in Figure 3. Additional forest plots on OSF (Supplemental Figures A15–A21) provide additional meta-analytical findings across domains. Studies consistently reveal severe psychosocial and psychopathological outcomes, including depression, sadness, shame, anxiety, stress, social isolation, post-traumatic stress disorder, suicidal ideation, and, in extreme cases, suicide attempts (e.g., Buelga et al., 2022; Figure 3). Being a digital hate target has also been linked to risky behaviors, such as delinquency or substance use (e.g., Biagioni et al., 2023), as well as lower academic performance, including reduced achievement and school attendance (e.g., Davis et al., 2014; Supplemental Figure A19). Additionally, social dynamics can function as predictors (e.g., negative peer influence being a risk factor) or outcomes (e.g., reduced relationship quality) following victimization (e.g., Fulantelli et al., 2022; Supplemental Figure A18). Other risk factors showing strong effect sizes are heavy use of communication technologies (e.g., Camerini et al., 2020; Supplemental Figure A17) as well as previous online and offline victimization (e.g., Caridade et al., 2019; Supplemental Figure A20). Notably, risk factors are reviewed more extensively than protective factors. Related to the latter, positive effects of interventions with respect to law, technology use, and awareness building have been documented, although effect sizes and success rates vary (e.g., Gaffney et al., 2019; Ng et al., 2022; Supplemental Figure A21). Overall, only a few studies reviewed coping strategies when confronted with the various facets of digital hate (e.g., psychological resources, confidence building, skill learning; e.g., Fernet et al., 2019). Finally, there is also a strong body of evidence about who is most vulnerable to digital hate. Women are more likely to experience digital hate (see OSF Supplemental Figure A15 for effect sizes), especially with respect to partnership and sexual hate (e.g., Fernet et al., 2019; Lozano-Blasco et al., 2023). When it comes to minority status, findings appear mixed with some reviews suggesting that minorities suffer from more victimization (e.g., Zych et al., 2019) and others showing no relationship (e.g., Zych et al., 2015). In some reviews, age was found to be a predictor of digital hate exposure; however, this appears to be inconclusive and seems to vary by culture (e.g., Lozano-Blasco et al., 2023). Further, some studies revealed that targets can switch roles and become perpetrators (e.g., Estévez et al., 2020).

The vast majority of reviews and meta-analyses focused on targets (see Table 2), followed by perpetrators (OSF Supplemental Figure A22 for a graphical overview). About half of all papers (*n* = 112; 54.4%; Supplemental Figure A23) examined more than one actor, with perpetrators and targets being most frequently studied (*n* = 108; 52.4%). Studies exploring perpetrators and audiences (*n* = 23; 11.2%) or targets and audiences (*n* = 23; 11.2%) were less common (Supplemental Figure A24). Only 21 reviews investigated all three actors simultaneously (10.2%).

The strong focus on perpetrators and targets is also evident when looking at the meta-analytical evidence (RQ3b). We sampled *n* = 39 meta analyses: Out of these, *n* = 27 (13.1%) considered perpetrators, six (2.9%) audiences, and *n* = 30 (14.6%) targets. An in-depth analysis of meta-analyses shows that 90% focused on adolescents, while only 18% dealt with adults (OSF Supplemental Table A13 and Figure A1). Additionally, the 39 meta-analyses investigated eight different digital hate concepts, making it challenging to draw meta-conclusions due to the risk of comparing apples and oranges (see Carpenter, 2020). A similar challenge arises with the wide array of over 100 variables examined across these meta-analyses, which were analyzed as predictors, outcomes, moderators, or mere associations. An overview of variables sorted by digital hate actor and their effect sizes can be found in the forest plots (Supplemental Figures A4–A21) on OSF. Overall, sociodemographic variables appear to be weak predictors, while strong associations are observed between digital hate perpetration and victimization, as well as between digital and offline hate. However, the mechanisms underlying these associations remain unclear. Mental health is consistently negatively linked with both perpetration and victimization, whereas the role of social factors (e.g., parental support) may have both positive and negative impacts. Furthermore, meta-analyses addressing environmental factors (e.g., work-related variables) are rare. When it comes to those studies that primarily looked at the contents and detection of digital hate, we found a great variety of automatic methods from natural language processing to identify digital hate, including topic modeling, machine learning, and deep learning. Such a heterogeneity of methodological approaches makes it difficult to compare detection methods across reviews.

As for research gaps (RQ4), there are remarkably consistent patterns across perpetrators, audiences, and targets. First, reviews point to an immense heterogeneity of terminologies, conceptualizations, and operationalizations (e.g., Caridade et al., 2019). For each part of the actor triad, there is a call for a greater harmonization of key terms to make studies comparable and arrive at more robust data on digital hate prevalence (e.g., Ma et al., 2019). Second, previous research has highlighted that most available studies come from North America and Europe, emphasizing the need for research in countries with different legal, economic, and socio-political backgrounds (most notably, Africa, Asia, and Latin America), ideally using comparative designs (Gini et al., 2018; Paz et al., 2020). Third, studies consistently identified a lack of both longitudinal and experimental studies (e.g., Marcum & Higgins, 2021; Windisch et al., 2022). Fourth, there is a consistent call for a greater variety in age groups (e.g., Fernet et al., 2019). More specifically, reviews conclude that much more research is needed using samples of people below 12, above 30, and particularly above 65 years of age (Marcum & Higgins, 2021). Also, reviews on both perpetrators and audiences consistently call for enhanced research efforts on interventions (e.g., Fulantelli et al., 2022). Finally, zooming in on digital hate content, there is a frequently voiced call for a unified hate detection framework (e.g., Yin & Zubiaga, 2021). Lacking comparability due to varying detection methods is consistently named a problem. Additionally, scholars frequently expressed a need to broaden the scope of scholarship by including non-Western countries and non-English languages (e.g., Paz et al., 2020).

## Discussion

The goal of this umbrella review was to synthesize existing reviews on digital hate. Reviewing a total of 206 reviews, our findings suggest several important directions for future research, regarding scope and methods, conceptualization, as well as actors and platforms. Before outlining key challenges in the field, it is important to highlight areas of consistent evidence. First, digital hate is strongly associated with a range of mental health consequences—including depression, anxiety, and self-harm—consistently reported for both victims and perpetrators. Second, gender differences are well documented, with women and girls more likely to experience digital hate. Third, common predictors of perpetration include low empathy, moral disengagement, antisocial tendencies, and heavy use of digital communication technologies. Fourth, studies often underscore the interplay between online and offline forms of hate, suggesting continuity across contexts. Finally, roles appear to be fluid; individuals may shift over time between being perpetrators, targets, and audience members. These converging findings offer a solid empirical foundation for future research.

### Conceptualization as a Key Challenge in Digital Hate Research

Despite these converging findings, substantial challenges remain. First, there is a lack of conceptual clarity. In fact, digital hate research may be considered a prime example of how jingles and jangles, clatters and clamors, as well as horizontal and vertical expansion (i.e., conceptual integration of qualitatively new and quantitatively milder phenomena, respectively; Haslam, 2016), can lead to a plethora of conceptually overlapping constructs. As a result of this conceptual diversity and evidenced by examining referential overlap across meta-analyses, extant literature shows an overwhelming heterogeneity of ostensibly incommensurable facets of digital hate that obscure overarching patterns across constructs, prevent cross-conceptual synergies, and complicate possible policy recommendations. There is a need for unifying conceptual work to disentangle the maze of terms, conceptualizations, and operationalizations. Instead of continuing to list disjunctive properties for supposedly distinctive phenomena, modular approaches embracing family resemblances as a gateway toward a conceptual core that is based on potential harmfulness (following Brown, 2017b) and, subsequently, distinguishing behaviors across meaningful dimensions (extending, e.g., Quandt, 2018) might be a promising compromise between lumping and splitting constructs (see Lawson & Robins, 2021) to consolidate this field. We argue that it might be instrumental for the field if researchers would work toward agreeing on dimensions on which digital hate can be situated such that, in the next step, labels (e.g., cyberbullying, online hate, cyberaggression) can be reassigned via parsimonious multi-dimensional specification (see, e.g., Wulff & Mata, 2023). Such a cross-disciplinary project needs a coordinated academic effort that goes beyond recent meritorious initiatives (e.g., Strippel et al., 2023); however, it might be necessary to restructure digital hate research in a more parsimonious direction.

Second, reviews point toward a need for standardized methods to detect and analyze digital hate content. The level of generalizability of detection models is low, validations across data sets, platforms, or topics are scarce, and studies seem to develop their own, unique methodology without refining or validating existing ones (Yin & Zubiaga, 2021). As content and its detection are deeply entangled, this umbrella review also highlights a pressing need for not only more research on content characteristics of digital hate but also systematic reviews and comparisons to aggregate these findings, making them more easily available to broader research communities. Similar to definitions and conceptualizations, methodological harmonization, particularly with respect to multi-country and -lingual studies, is necessary.

### Research Scope and Methods

Third, research on different digital hate facets (e.g., cyberbullying, online hate speech) has failed to generate solid knowledge across age groups. More specifically, adolescents and young adults are studied far more frequently than children and adults. This narrow emphasis on these two age groups may be explained by the dominance of reviews on cyberbullying and related concepts in the school context. Although the impact of developmental levels for digital media use and effects is well understood and has been theorized prominently (see, e.g., Valkenburg & Peter, 2013), reviews largely turned out unable to compare several age groups.

Fourth, reviews consistently noted that most currently published evidence comes from Western countries such as the United States, Germany, or Canada. This finding was also partly mirrored in the authorship of the analyzed reviews. There is a consensus that digital hate needs to be studied in regions with different legal, cultural, religious, and social conditions. Fifth, many reviews called for more experimental and longitudinal evidence. As in any field, compared to the wealth of cross-sectional research, such studies are necessary to test for causality, examine directionality of relationships, and specify long-term impacts.

It is important to note that these five shortcomings appear to apply to the entire body of digital hate research. Consequently, the level of generalizability appears low despite thousands of studies. In terms of practical implications, it is therefore difficult to arrive at nuanced recommendations for policymakers, or tech companies. Of note, the added value of yet another single-platform, Western-country, and single-age-group study can be questioned. There needs to be a major shift in the research agenda in the field of digital hate.

### Actors and Platforms

In addition to these well-known problems, our findings suggest some novel issues. To begin with, the state of the systemizing evidence across different actors appears uneven. There are a lot of reviews on perpetrators and targets. It is safe to say from these reviews that digital hate is very much prevalent and that it can have devastating effects on individuals (i.e., mostly adolescents and young adults in Western countries). However, less systematic evidence is available regarding coping strategies, attitudes and beliefs, mechanisms behind digital and offline hate transitions, environmental effects (such as work contexts or platform affordances), roles of emotions, and specificity of social dynamics, especially for digital hate concepts beyond cyberbullying. Compared to perpetrators and targets, reviews on audiences of digital hate were much less frequently published, particularly concerning interventions aiming to diminish bystander apathy. This pattern holds for meta-analytical evidence as well, with fewer meta-analyses on audiences compared to perpetrators and targets. Additionally, due to conceptual heterogeneity, effect sizes between digital hate types are difficult to compare. Systematically investigating specific mechanisms across concepts would help clarify their comparability. Further, we find that the majority of reviews on digital hate do not analyze platform differences. In fact, most research deals with single platforms, and currently available reviews do not sufficiently examine how different platform characteristics affect the emergence and/or inhibition of digital hate (e.g., recommender systems, content moderation, anonymity). Considering that user behaviors are significantly shaped by platform affordances (see Sude & Dvir-Gvirsman, 2023), this lack of cross-platform research is problematic. Such an endeavor requires interdisciplinary research (i.e., social and computational sciences) at various language and content levels, but also user characteristics or network approaches might be important. By offering a comprehensive view, cross-platform research ensures that policy responses are not only reactive but also proactive, anticipating and mitigating threats.

Finally, approximately half of all reviews investigated more than one actor (most often, perpetrators and targets). Perpetrators and audiences, as well as targets and audiences, were less likely to be simultaneously analyzed. Reviews examining all three actors simultaneously are scarce. We believe this lack of cross-actor research is another major shortcoming. Perpetrators, audiences, and targets are not fixed roles. That is, a perpetrator can become an audience member or a target, and likewise, a target can also become a perpetrator or a bystander (e.g., Lambe et al., 2019). Only analyzing single actors, or actor tandems, combined with a lack of longitudinal research, cannot lead to a proper understanding of what likely are complex and dynamic interrelations across the actor triad. A similar argument can be made for content. The perception of a single instance of hateful content likely differs significantly between perpetrators, audience members, and targets; however, it may also be relevant to consider how often people have been in each of the actor roles in the past. What is hateful content to a member of a minority group may not appear hateful to others. If varying perceptions on digital hate are not considered, then the key question of what is treated (and coded) as hateful content (and what is not) is completely in the hands of those who write the codebook or train the data. Previous research suggests that there can be bias against particular forms of hate (Binns et al., 2017). The analysis of digital hate content needs to be associated with the perception of hate by several actors, most notably, the targets.

### Limitations

Some limitations must be noted. First, our umbrella review only included studies published in English. There is a significant stream of research in countries that do not have English as their first language. Our analysis may thus overrate potential biases in terms of authorship. Second, we included narrative reviews, which may be seen as lower in quality due to their lack of systematization. However, they were still valuable for this umbrella review, as most research questions focused on variables independent of paper quality (e.g., concept distribution or WEIRD bias). Narrative reviews also offer flexibility in the research process, which we considered beneficial for providing a broad overview. Third, while we looked at the scope, actors, definitions, main findings, and research gaps of reviews about digital hate, we were unable to code and analyze more detailed information, such as the majority or minority status of analyzed groups. Also, we did not attempt to statistically synthesize meta-analyses by calculating effect sizes at a meta level (i.e., a meta meta-analysis). Finally, one may criticize that we sampled heterogeneous concepts, that is, comparing “apples and oranges.” In fact, the disadvantage of an umbrella review is that it remains on an abstract level, leading to overly broad conclusions. More specific reviews of sub-fields of digital hate are therefore warranted. Also, we looked at the key actors involved in digital hate: perpetrators, audiences, and targets. Platform policy makers, content moderators, or other decision-making actors that impact the spreading of digital hate were not explicitly addressed. However, we argue that different facets of digital hate are by no means independent; instead, they most likely overlap or co-occur substantially (i.e., they are more like different types of apples). All forms of hate included in this umbrella review share the notion of digitally transmitted malicious behavior toward other individuals or groups, and understanding drivers and boundary conditions for such behaviors on a broad scale is key for modern society. In contrast, a selection of single concepts may be even more problematic (and somewhat arbitrary) as boundaries to neighboring concepts are not clear-cut. It thus makes sense to evaluate the broader body of research with an umbrella review instead of continuing to examine mosaic bits of a very similar kind.

## Conclusion

We conclude that there is a need to restructure and harmonize digital hate research. Similar terms are used for different concepts, and there is generally a lack of operational consistency, hampering progress in this area of scholarship. Furthermore, more systematic evidence is needed with respect to platform differences, the role of age, experimental and longitudinal research methodology, as well as country differences. Finally, we call the field to systematically analyze digital hate across perpetrators, audiences, and targets in order to arrive at a more dynamic understanding of how actor roles may shift over time.
